# Crystal structure of the N-terminal domain of human CDC73 and its implications for the hyperparathyroidism-jaw tumor (HPT-JT) syndrome

**DOI:** 10.1038/s41598-017-15715-9

**Published:** 2017-11-15

**Authors:** Wei Sun, Xiao-Lin Kuang, Yan-Ping Liu, Li-Fei Tian, Xiao-Xue Yan, Wenqing Xu

**Affiliations:** 1 National Laboratory of Biomacromolecules, CAS Center for Excellence in Biomacromolecules, Institute of Biophysics, Chinese Academy of Sciences, Beijing, 100101 P. R. China; 2 College of Life Sciences, University of Chinese Academy of Sciences, Beijing, 100049 China; 3 Department of Biological Structure, University of Washington, Seattle, Washington, 98195 USA

## Abstract

CDC73/Parafibromin is a critical component of the Paf1 complex (PAF1C), which is involved in transcriptional elongation and histone modifications. Mutations of the human *CDC73/HRPT2* gene are associated with hyperparathyroidism-jaw tumor (HPT-JT) syndrome, an autosomal dominant disorder. CDC73/parafibromin was initially recognized as a tumor suppressor by inhibiting cell proliferation via repression of *cyclin D1* and *c-myc* genes. In recent years, it has also shown oncogenic features by activating the canonical Wnt/β-catenin signal pathway. Here, through limited proteolysis analysis, we demonstrate that the evolutionarily conserved human CDC73 N-terminal 111 residues form a globularly folded domain (hCDC73-NTD). We have determined a crystal structure of hCDC73-NTD at 1.02 Å resolution, which reveals a novel protein fold. CDC73-NTD contains an extended hydrophobic groove on its surface that may be important for its function. Most pathogenic *CDC73* missense mutations associated with the HPT-JT syndrome are located in the region encoding CDC73-NTD. Our crystal and biochemical data indicate that most CDC73 missense mutations disrupt the folding of the hydrophobic core of hCDC73-NTD, while others such as the K34Q mutant reduce its thermostability. Overall, our results provide a solid structural basis for understanding the structure and function of CDC73 and its association with the HPT-JT syndrome and other diseases.

## Introduction

Human CDC73 (hCDC73), also known as parafibromin, is a human nuclear protein encoded by the cell division cycle 73 (*CDC73*) gene which is located on chromosome 1q31.2^1,2^. Mutations of human *CDC73* are associated with hyperparathyroidism-jaw tumor (HPT-JT) syndrome, which is an autosomal dominant disorder characterized by parathyroid tumors, fibro-osseous jaw tumors, cystic kidney lesions and uterine tumors^1,3–5^. *CDC73* mutations are also associated with parathyroid carcinomas and renal tumors^6–10^ (Supplementary Table S1). CDC73 is a core subunit of the polymerase-associated factor 1 complex (PAF1C) that binds to RNA polymerase II^11–13^. PAF1C participates in recruitment of histone modification factors and promotion of transcriptional elongation^14–16^.

Human CDC73 was initially proposed as a tumor suppressor which can repress expression of cyclin D1^17,18^ and c-myc proto-oncogene^19^. For example, CDC73 can recruit the histone methyltransferase (HMTase) SUV39H1 to the *cyclin D*1 gene to inhibit its expression by inducing methylation of H3 K9^18^. Loss-of-function mutations of hCDC73 can thus lead to overproduction of cell cycle control proteins and promote cell proliferations. However, in some other occasions, CDC73 possesses oncogenic features. In particular, it can serve as a transcriptional coactivator of the Wnt/β-catenin signaling pathway by directly interacting with β-catenin in the nucleus^20^. This interaction can be stabilized by SHP2-mediated dephosphorylation of CDC73 residues Y290, Y293 and Y315 in the nucleus^21^. Recent studies also show that CDC73 can act as a scaffold protein to coordinate Wnt/Hedgehog/Notch pathway and plays important roles during embryogenesis and organogenesis^22,23^. Both tumor-suppressing and oncogenic effects of CDC73 rely on the N-terminal portion of hCDC73^18,20^.

The C-terminus of hCDC73 (residues 357–531) bears a conserved Ras-like domain homologous to yeast CDC73, which promotes the interaction between the PAF1 complex and chromatin^24^. Crystal structures of the C-terminal domain of yeast CDC73 have been reported^24,25^, but there is no 3D structural information of the hCDC73 N-terminal region to date. In addition to the β-catenin interaction domain (CID, residues 218–263), the hCDC73 N-terminal region also contains a functional nuclear localization signal (residues 125–139)^26^, as well as three proposed nucleolar localization signals^27,28^. Most of the documented *CDC73* mutations are nonsense truncation mutations which result in physiologically inactive proteins^3^ (Supplementary Table S1). Although a number of missense variants of hCDC73 have been found in HPT-JT and other diseases, only a few of them, including K34Q, I60N, L64P and R91P, have been shown to be pathogenic (Supplementary Table S1). Strikingly, all of them are located in the N-terminal region of CDC73, underlying the functional importance of this region^17,29,30^. For example, I60N shows decreased protein stability and a loss of its ability to down-regulate c-myc expression, and is associated with ossifying fibroma^30^. The L64P mutation found in HPT-JT patients was found to be able to retain nuclear expression and form PAF1C, but showed reduced interaction with the Set1 histone methyltransferase complex and decreased its HMTase activity^17,31^.

To understand how the CDC73 N-terminal domain contributes to CDC73 functions and how CDC73 pathogenic mutations may lead to HPT-JT and other diseases, we have characterized the structural features of the CDC73 N-terminal regions and revealed a previously unidentified N-terminal globular domain. Furthermore, we have determined a crystal structure of CDC73-NTD at ultrahigh 1.02 Å resolutions and characterized CDC73 pathogenic missense mutants using biochemical assays. Our study provides novel insights into CDC73 function and molecular mechanisms of HPT-JT mutants.

## Results

### N-terminal half of hCDC73 contains a conserved and protease-resistant domain

**Figure 1:**
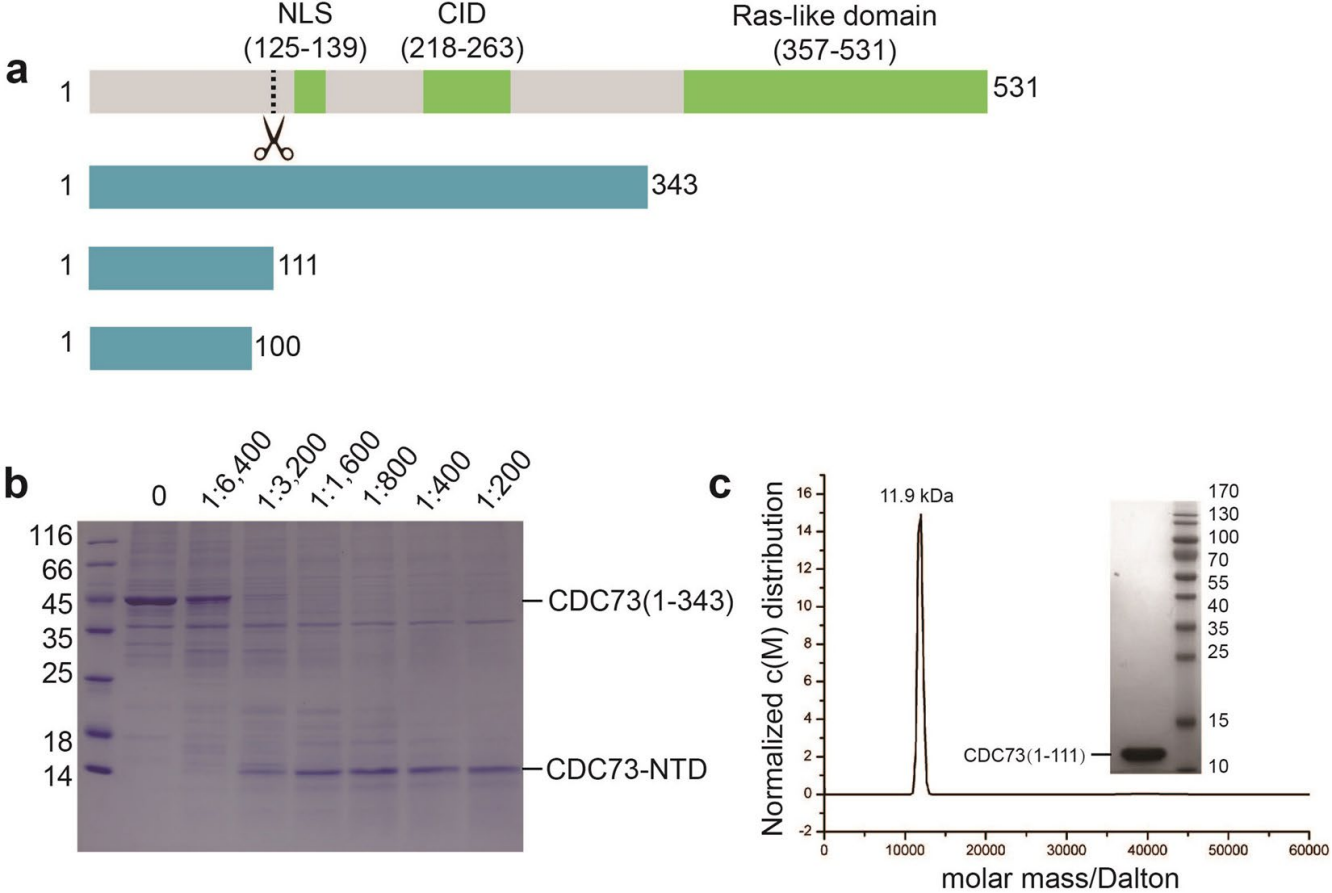
Biochemical characterization of the N-terminal domain of CDC73 (CDC73-NTD). (**a**) Schematic representation of human CDC73 and CDC73 fragments used in this study. Full-length human CDC73 consists of 531 residues containing a nuclear localization signal (NLS) and a β-catenin interaction domain (CID). The dash line indicates the boundary of a protease-resistant fragment. (**b**) One trypsin-resistant fragment can be obtained through limited proteolysis of CDC73(1–343), which contains the first 111 residues of hCDC73 (CDC73-NTD). The marked ratios are the molar ratios of trypsin over hCDC73(1–343) used in limited proteolysis. The SDS-PAGE gel was stained with Coomassie Brilliant Blue. (**c**) Analytical ultracentrifugation (AUC) analysis demonstrates that CDC73(1–111) has a molecular weight of 11.9 kDa, which is close to the nominal MW of monomeric hCDC73(1–111) (12.7 kDa). The inlet shows the SDS-PAGE of our hCDC73(1–111) sample used for crystallographic and AUC analysis, stained with Coomassie Brilliant Blue.

To understand how parafibromin is involved in β-catenin-mediated Wnt signal activation, we tested the overexpression and purification of numerous hCDC73 fragments. We found that human CDC73(1–343), which contains β-catenin interaction domain of CDC73 (CID) and the nuclear localization signal (NLS, Fig. 1a), can be purified in decent quantities. However, during purification, hCDC73(1–343) was susceptible to degradation and robustly led to a stable ~12kD fragment. Thus we performed limited proteolysis of hCDC73(1–343) with trypsin and consistently observed one stable fragment (Fig. 1b). LC-MS/MS analysis revealed that this protease-resistant fragment contains at least the first N-terminal 100 residues and likely the first 111 residues of hCDC73. We thus named this protease-resistant region as hCDC73-NTD. Based on sequence conservation and the size of trypsin-resistant fragment, we generated new plasmids and purified both hCDC73(1–100) and hCDC73(1–111). SEC and analytical ultracentrifugation (AUC) analysis clearly show that both fragments are monomeric in solution (Fig. 1c).

### Crystal structures of human CDC73-NTD

**Figure 2:**
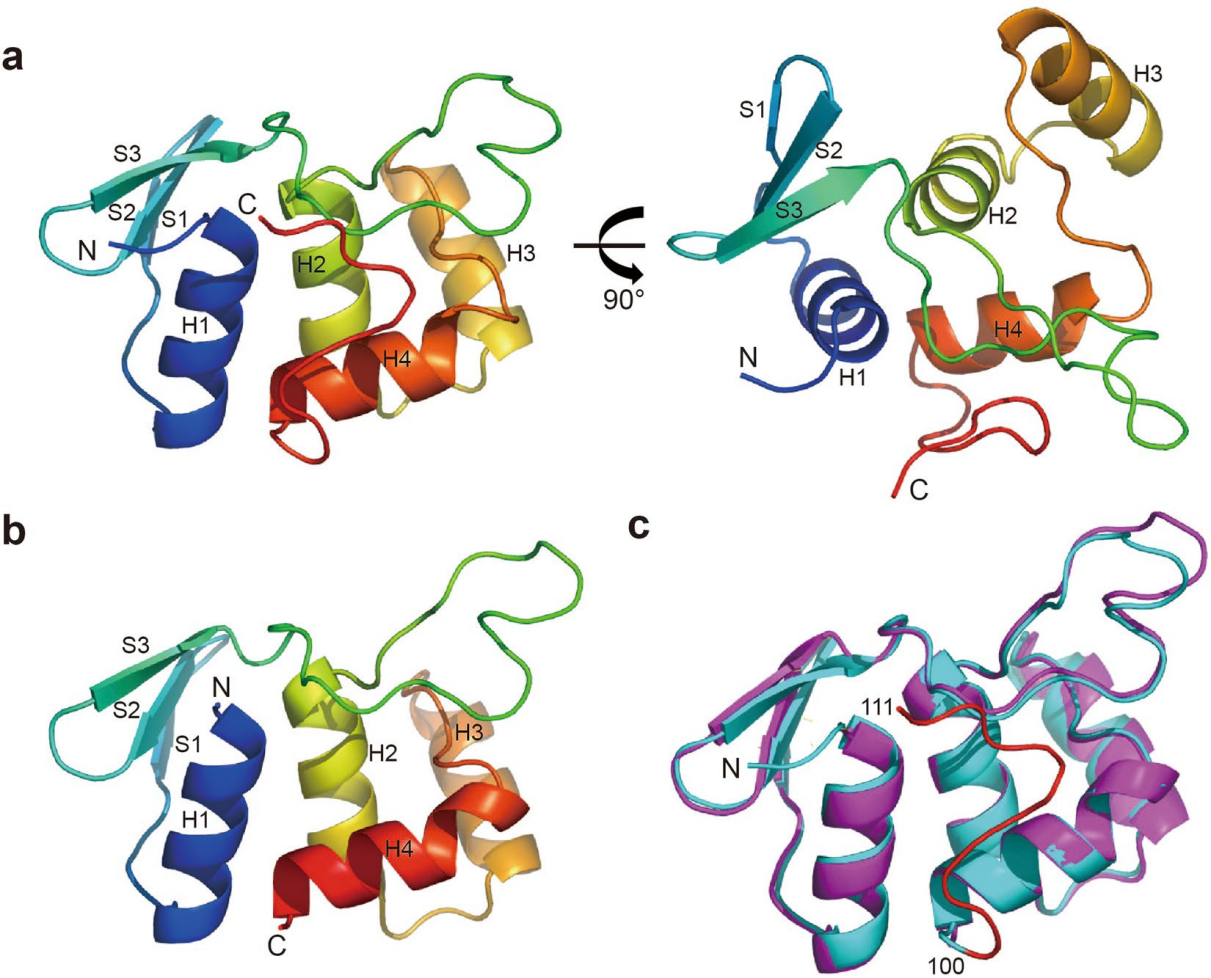
Overall structure of CDC73-NTD. (**a**) Two orthogonal views of the crystal structure of CDC73(1–111). (**b**) Overall structure of CDC73(1–100). (**c**) Superposition of CDC73(1–111) and CDC73(1–100) crystal structures. CDC73(1–111) and CDC73(1–100) are shown in cyan and magenta, respectively. The red loop represents the extra 11 C-terminal residues of CDC73(1–111).

Since hCDC73-NTD is evolutionarily conserved and harbors most of the documented pathogenic CDC73 missense mutations, we determined crystal structures of hCDC73-NTD. First, we determined the crystal structure of CDC73(1–100) using the SAD method (with a soaked platinum derivative) at 1.4 Å resolution, and then the crystal structure of CDC73(1–111) at 1.02 Å resolution (Table 1; Fig. 2a,b). There are one and two molecules in each asymmetric unit for CDC73(1–111) and CDC73(1–100) crystals, respectively. The model corresponds well to the electron density map (Supplementary Fig. S1). The overall structure of CDC73(1–111) is almost identical with CDC73(1–100), with a r.m.s.d. of Cα positions being 0.894 Å. Both CDC73(1–100) and CDC73(1–111) fold into a globular domain. The extra 11 residues of CDC73(1–111) pack tightly on surface of the CDC73(1–100) core structure (Fig. 2c). Analysis using the DALI server shows that CDC73(1–100) has a novel fold (Supplementary Table S2). This domain structure is composed of four α helices and one anti-parallel β-sheet consisting of three β-strands (Fig. 2b).

**Table 1:** Summary of crystallographic statistics.

Data collection	CDC73(1–100) - native	CDC73(1–100) - derivatives	CDC73(1–111) - native
Space group	*P2*_1_	*P*2_1_	*P*2_1_2_1_2_1_
Cell dimensions			
A, b, c (Å)	28.59, 53.50, 65.73	28.55, 53.52, 66.01	38.09, 43.68, 67.63
α, β, γ (°)	90.00, 95.66, 90.00	90.00, 95.73, 90.00	90.00, 90.00, 90.00
Wavelength (Å)	0.9782	0.9782	0.9782
Resolution (Å)	50–1.40 (1.42–1.40)	50–1.40 (1.42–1.40)	50–1.02 (1.05–1.02)
R_merge_ (%)	4.3 (18.1)	11.5 (57.4)	12.2 (35.9)
I/σI	41.92 (10.0)	72.38 (6.5)	38.61 (4.9)
CC1/2	0.996 (0.986)	0.989 (0.964)	0.964 (0.891)
Completeness (%)	98.9 (99.1)	99.0 (99.5)	99.0 (79.8)
Redundancy	6.6 (6.3)	25.3 (18.8)	6.3 (5.8)
Wilson B factors (Å^2^)	11.0	15.9	9.2
Refinement			
Resolution (Å)	19.5–1.40		19.4–1.02
No. Reflections	38451		57501
R_work_/R_free_ (%)	13.52/17.42		11.94/13.94
No. atoms	1661		1740
No. of water	348		284
Average B factors (Å^2^)			
Protein	15.37		13.18
H_2_O	33.25		32.27
R.m.s deviations			
Bond lengths (Å)	0.012		0.011
Bond angles (°)	1.361		1.516
Ramachandran plot (%)			
Most Favorable	100.0		100.0
Allowed	0.0		0.0
Outliers	0.0		0.0

### Analysis of pathogenic mutations in human CDC73-NTD

**Figure 3:**
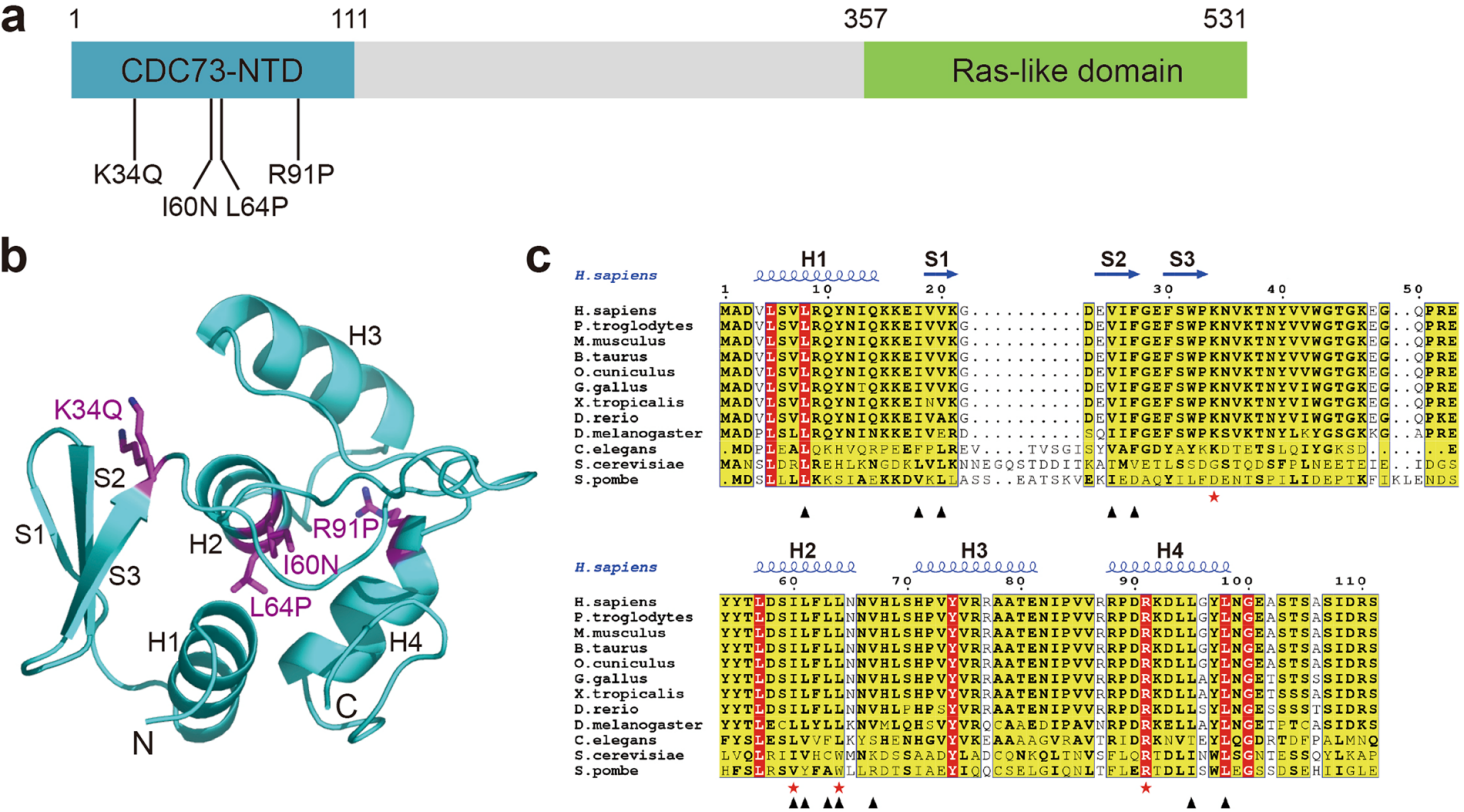
CDC73-NTD harbors all reported pathogenic missense mutations. (**a**) Domain structure of CDC73 shows that four characterized pathogenic mutations are clustered in CDC73-NTD. (**b**) Positions of those four pathogenic missense mutations are labeled in the structure of CDC73-NTD. (**c**) Sequence alignment of CDC73(1–111) from ten species shows that CDC73-NTD is evolutionarily conserved from yeast to human. The  indicate positions of CDC73 pathogenic missense mutations. The ▴ mark residues forming the hydrophobic groove of CDC73-NTD.

Most pathogenic mutations of human CDC73 are nonsense or frame-shift mutations (Supplementary Table S1). However, at least four missense mutations have been characterized (Fig. 3a). They are K34Q, I60N, L64P, R91P^6,29–31^. Mutations L64P and R91P, occurring at residues located in the helices H2 and H4, respectively, are helix-disruptive and may thus destroy the CDC73-NTD folding (Fig. 3b). Ile60 forms part of the hydrophobic core of CDC73-NTD. The I60N mutation would cause steric clashes in the CDC73-NTD hydrophobic core and thus disrupt its structure (Fig. 3b). To examine the biochemical properties of these mutants, we tested overexpression and purification of several human CDC73(1–111) missense mutants in *E. coli*, including K34Q, I60N, L64Pand R91P. Almost all of them, including I60N, L64P, and R91P, are essentially insoluble in *E. coli*, consistent with our structure-based prediction that these missense mutations disrupt the structure of CDC73-NTD.

### Characterization of the K34Q mutant and the role of the hCDC73(1–111) tail

**Figure 4:**
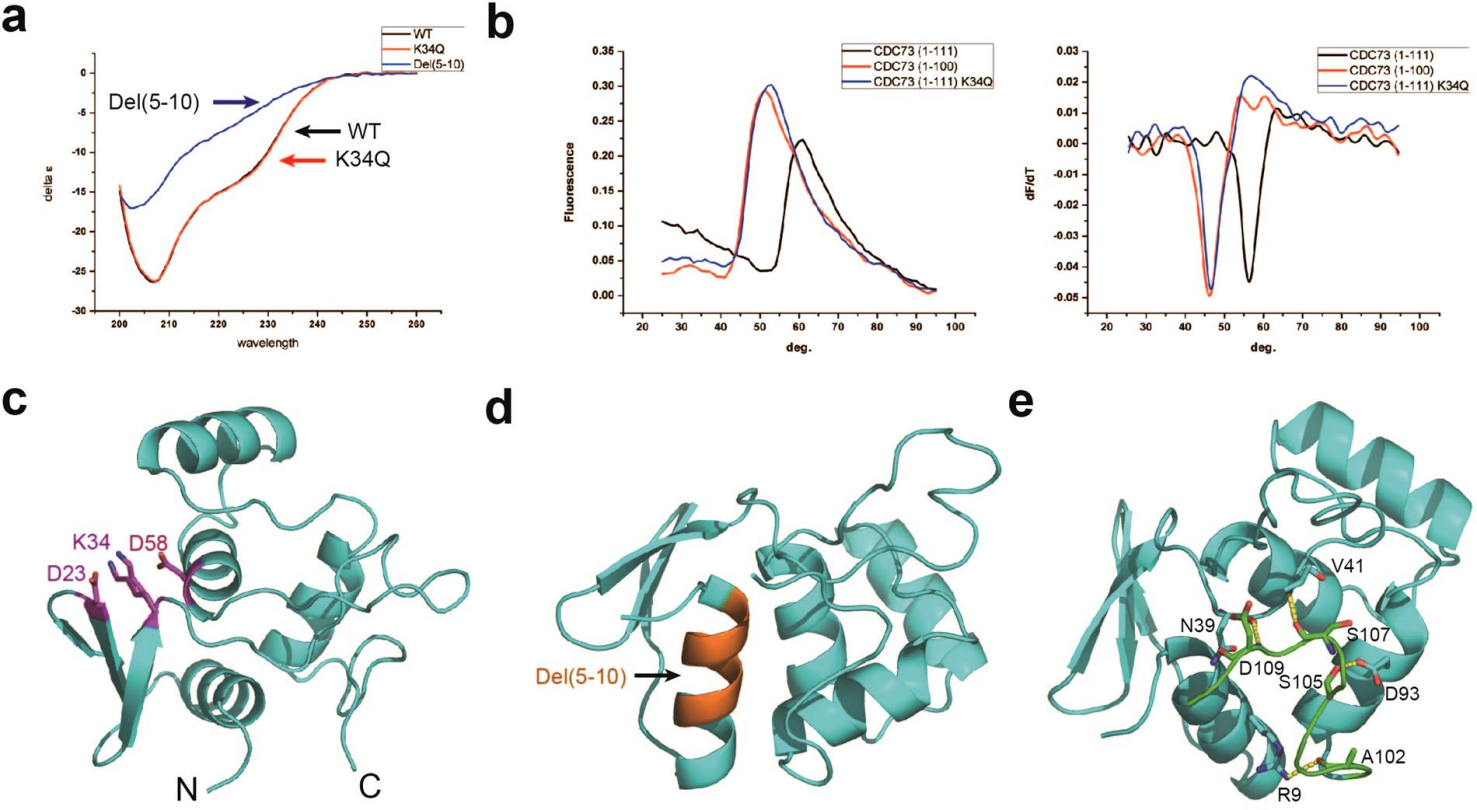
Characterization of the CDC73 K34Q mutant and analysis of the differences between CDC73(1–111) and CDC73(1–100). (**a**) CD spectra of the CDC73(1–111) K34Q mutant, with wild-type (WT) and the del(5–10) mutant as positive and negative controls, respectively. (**b**) Thermofluor assays reveal a T_m_ difference of 10 °C for both WT vs K34Q CDC73(1–111) and CDC73(1–111) vs CDC73(1–100). The left panel shows the fluorescence change corresponding to temperature. The right panel is the calculation of melting temperatures. (**c**) In our crystal structure, the K34 sidechain (with dual conformations observed) forms salt bridges with D23 and D58. (**d**) Positions of residues 5–10 in the CDC73 H1 helix, shown in orange in the structure. Internal deletion of these 6 residues disrupts the overall fold of hCDC73-NTD. (**e**) The CDC73-NTD C-terminal tail (residues 101–111) interacts with the rest of CDC73-NTD via hydrogen bonds and van der Waals forces. CDC73(1–100) and the C-terminal tail are shown in cyan and green, respectively. The hydrogen bonds between them are labeled with yellow dash.

Among CDC73 missense mutants we tested, K34Q was the only one which we could produce its soluble form in decent amount for further characterization. To test whether the K34Q mutation changed the secondary structure of CDC73-NTD, the CD spectra of wild-type (WT) and K34Q mutant forms of CDC73(1–111) were measured. Not surprisingly, the overall secondary structure compositions are essentially identical (Fig. 4a). In contrast, the del(5–10) mutant of hCDC73(1–111), with deletion of residues 5–10 in helix 1 (H1), could be purified with a very low yield and demonstrated a CD spectrum of a largely unfolded protein (Fig. 4a,d). To examine whether the K34Q mutation changes its thermostability, we measured the melting temperature of WT and K34Q using a Thermofluor assay. Apparently, the K34Q mutant (T_m_ = 46 °C) is thermodynamically less stable than the WT human CDC73(1–111) (T_m_ = 56 °C) (Fig. 4b). In our crystal structure, the Lys34 sidechain can adopt two conformations, both forming salt bridges with adjacent Asp23 and Asp58. The K34Q mutant may thus destabilize the CDC73-NTD structure by disrupting these salt bridges (Fig. 4c).

This C-terminal 11 residues of CDC73(1–111) interact extensively with the CDC73(1–100) core, which may stabilize the overall structure (Fig. 4e). Indeed, our Thermofluor assay showed that hCDC73(1–100) and hCDC73(1–111) have T_m_ values of 46 °C and 56 °C, respectively (Fig. 4b).

### A hydrophobic groove on the CDC73-NTD surface and the NoLS

**Figure 5:**
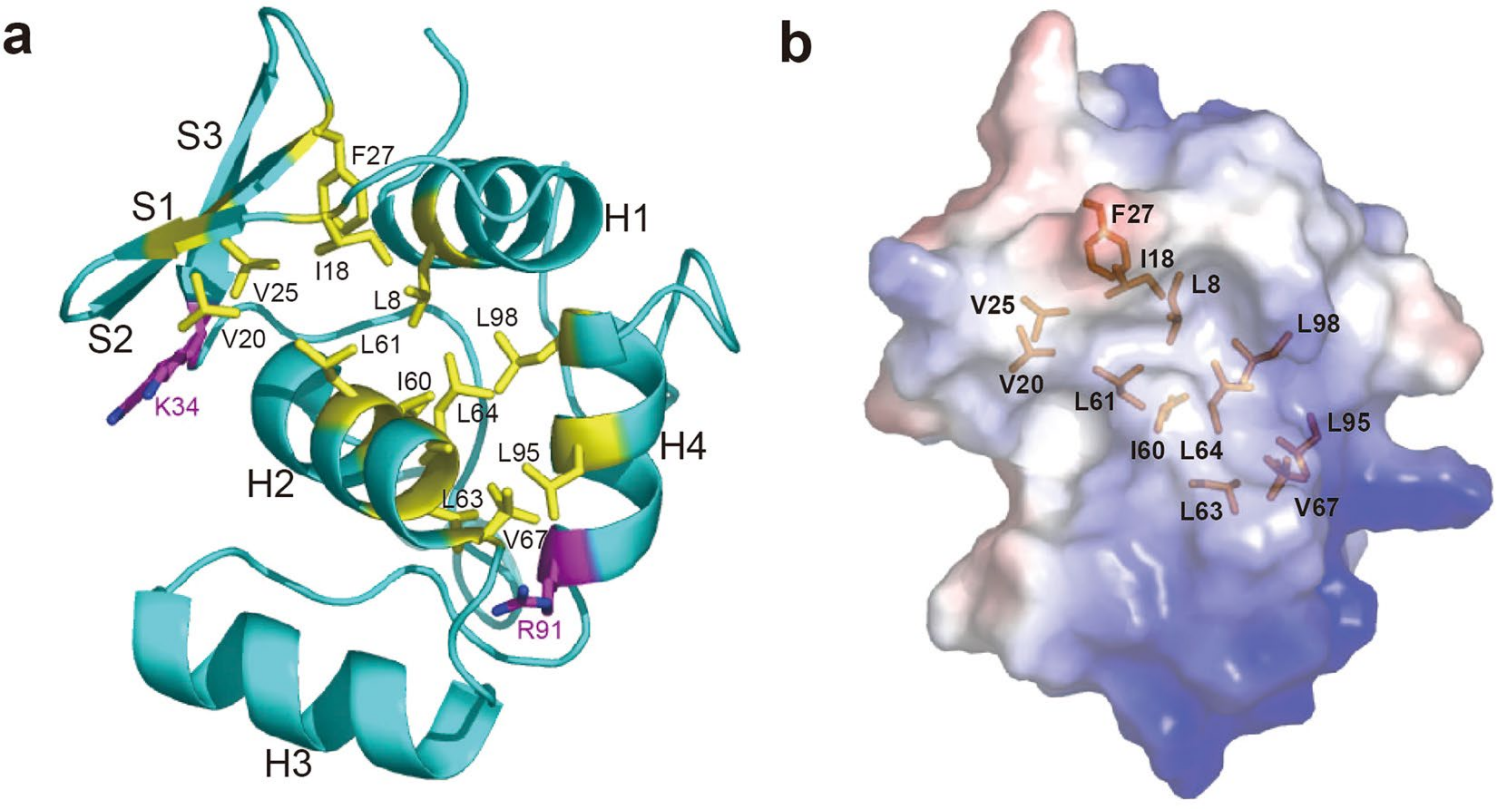
A hydrophobic groove on the surface of CDC73-NTD. (**a**) Residues composing the hydrophobic groove are shown as yellow sticks. Two residues associated with CDC73 pathogenic mutations, K34 and R91, are located at two ends of the hydrophobic groove. (**b**) Electrostatic potential surface map was shown with the same orientation as the structure in panel a. Residues forming the hydrophobic groove are shown in sticks.

Human CDC73-NTD has a hydrophobic groove on the surface. It is formed by CDC73-NTD β-strand S1 and helices H1, H2 and H4 (Fig. 5a and Supplementary Fig. S2). Hydrophobic residues involved in this groove, L8, I18, V20, V25, F27, I60, L61, L63, L64, L67, L95 and L98, are mostly conserved among CDC73 proteins from yeast to human, suggesting a conserved structural feature that may have functional implications (Fig. 3c). Interestingly, pathogenic mutations I60N and L64P are located inside of this groove, while the other two pathogenic mutants K34Q and R91P are located on two ends of this groove. Although the function of this groove remains unclear, it is likely that the deformation of this groove is associated with CDC73 malfunction.

A separate issue regarding CDC73-NTD is a proposed nucleolar localization signal (NoLS), which is among the three proposed NoLS’s that mediate the nucleolar localization of hCDC73^27^. In our crystal structure, this NoLS (residues 76–92) spans helices H3, H4 and the H3-H4 loop, and forms an exposed surface of CDC73-NTD that is adjacent but distinct from the hydrophobic groove (Supplementary Figs S2 and S3). This proposed NoLS appears conserved among species and may be important for CDC73 functions, since three HPT-JT associated mutants, R77P, del(NIP) and del(VV) have been identified and characterized within this NoLS^28^. There is no general mechanism how NoLS promotes nucleolar localization^32,33^, and it remains unclear how this proposed CDC73 NoLS works.

## Discussion

HPT-JT, parathyroid carcinomas and renal tumors are clinically severe cases and threat many human lives. Discovery of pathogenic CDC73 mutants, especially pathogenic missense mutations observed in CDC73-NTD, strongly demonstrate the biological and medical significance of the CDC73 N-terminal domain. It remains unclear how CDC73 exerts its biomedical effects. Recently, a mouse model for HPT-JT with Cdc73 depletion, showing parathyroid tumors and uterine neoplasms, has been established and will be helpful for future research on these tumors^34^.

Here, we demonstrate that CDC73-NTD can form a well-folded globular domain, and is conserved from human to yeast. Most of the reported CDC73 missense pathogenic mutations are clustered in this region. Our limited proteolysis data suggests that the region between the well folded CDC73-NTD (residues 1–111) and the Ras-like C-terminal domain (residues 357–531) is largely unfolded, but is involved in interactions with a number of CDC73 binding partners including β-catenin^18,20,23,35^. Previous works showed that most *HRPT2* mutations (Supplementary Table 1) would result in truncation of the CDC73 protein^3^. Lack of the C-terminal Ras-like domain may cause deficiency in histone ubiquitination and methylation^36,37^. Some truncations lacking the region interacting with other Paf1 complex subunits would impair the formation of this complex^18^. CDC73 is predominantly a nuclear protein, and is rich in nucleoli^26,27,38^. Although it remains unclear how the proposed NoLS in CDC73-NTD works, the association of CDC73 mutations within this NoLS with HPT-JT suggests biological significance of this NoLS.

The pathogenic CDC73 K34Q mutant was identified from patient with renal tumors. The K34Q mutant is localized to the nucleus and can effectively participate in PAF1C formation^6^. Unlike other mutants, the CDC73(1–111) K34Q mutant is as soluble as the WT protein. Thermofluor assay shows that the melting temperature of K34Q is 10 °C lower than that of WT. This suggests that the K34Q mutant may lose the ability to suppress expression of *cyclin D* *1* due to lower thermal stability. Alternatively, since Lys34 is located at one end of the hydrophobic groove and may be involved in interaction with other CDC73 binding partner(s), the K34Q mutation may lead to functional loss by abolishing these CDC73-partner interaction(s).

In summary, we reveal a well-folded and conserved N-terminal domain of CDC73. Through crystallographic analysis, we provide solid structural basis for understanding CDC73 pathogenic mutants within this domain. We suggest that most, if not all, CDC73 pathogenic mutations lead to loss of CDC73 functions by disrupting the folding or thermostability of CDC73-NTD. The observation of a hydrophobic groove on CDC73 surface will also help with understanding the function of this conserved domain.

## Materials and Methods

### Cloning, purification and crystallization

The full-length human CDC73 gene was cloned from a cDNA library, and constructed into pET28a vector. Distinct fragments were also inserted into a pET28a vector. Mutant plasmids of hCDC73 were constructed with the Stratagene Quickchange Kit.

Constructed plasmids were transformed into BL21(DE3) for protein expression. Cell cultures were induced with 0.2 mM IPTG for 20 h at 18 °C when OD_600_ reached roughly 0.8. Cell pellets were disrupted by sonication. Afterwards cell pellets were spun down at a speed of 30,700 g. CDC73 proteins were purified by a sequential procedure: nickel column, his-tag cleavage by TEV protease, second nickel column, ion-exchange column and size exclusion column. Purified proteins were stored in a buffer containing 20 mM Tris pH7.5, 400 mM NaCl and 4 mM DTT.

After purification, proteins were concentrated to 10 mg/ml and used for crystallization screens. When an initial hit was observed, optimization was conducted employing the hanging-drop method. The growth condition of CDC73(1–100) crystal is 0.2 M MgCl_2_, 0.1 M Tris pH8.5, 25% PEG3350, and CDC73(1–111) crystals were obtained in 0.1 M Tris pH8.5, 25% PEG3350.

### Data collection and structure determination

The native X-ray diffraction data sets of CDC73(1–100) and CDC73(1–111), as well as the potassium tetranitroplatinate-derivative data set of CDC73(1–100), were collected at the BL19U beam line in Shanghai Synchrotron Radiation Facility (SSRF). All data sets were processed with HKL3000^39^. The phase problem of CDC73(1–100) was solved by single anomalous dispersion (SAD) method using Autosol in PHENIX^40^, and autobuilding was done with ARP/wARP in CCP4 suite^41^. Model building was performed with COOT^42^ and iterative cycles of refinement were done with Refmac5 in CCP4 suite^41^ and PHENIX^40^. Thereafter, the structure of CDC73(1–111) was solved by molecular replacement using the Phaser in PHENIX, with our CDC73(1–100) crystal structure as the template. Due to the ultrahigh resolution of CDC73(1–111) crystals, we added alternative conformations and refined occupancy for each conformation during refinement. Anisotropic B factor refinement was used. Finally, hydrogen atoms were added to the structure. All structural pictures were generated using PyMOL^43^. Crystallographic statistics are shown in Table 1.

### Limited proteolysis and mass spectrometry

His-CDC73(1–343) was digested with trypsin at different molar ratios of trypsin:CDC73 (1:6,400, 1:3,200, 1:1,600, 1:800, 1:400 and 1:200). The incubation buffer is 20 mM Tris pH7.5, 400 mM NaCl. Samples were incubated at room temperature for 1 h. After that protein loading buffer was added and boiled at 100 °C for 10 min for SDS-PAGE. The degraded fragment after limited proteolysis was cut from the SDS-PAGE gel and sent for LC-MS/MS analysis in the Laboratory of Proteomics, Institute of Biophysics, CAS.

### Analytical ultracentrifugation, CD spectrum and thermofluor assay

Sedimentation velocity experiment was performed with a Beckman ProteomeLab XL-I analytical ultracentrifuge at 20 °C. Proteins were prepared in a buffer containing 20 mM Tris pH7.5, 400 mM NaCl, with A_280_ absorbance of ~0.7. Data collection was performed at 60krpm at a wavelength of 280 nm. Finally, interference sedimentation coefficient distribution (c(M)) was calculated using software SEDFIT.

Circular dichroism was performed using a ChirascanPlus CD spetrometer. The protein buffer was changed to a phosphate buffer (137 mM NaCl, 2.7 mM KCl, 10 mM Na_2_HPO_4_, 2 mM KH_2_PO_4_) before the experiment, and proteins were concentrated to 0.2 mg/ml.

Melting temperatures of the wild-type, K34Q and del(5–10) mutants of CDC73(1–111) were measured using quantitative PCR machine Rotor-Gene 6600. Before experiment, proteins were concentrated to 4 mg/ml, and then 18 μl PBS was mixed with 1 μl protein and 1 μl SYPRO^TM^ ORANGE dye. The temperature gradient was set to a range from 25 °C to 95 °C with 1°/min increment.

### Data availability

Atomic coordinates and structure factors of hCDC73(1–100) and hCDC73(1–111) have been deposited in the Protein Data Bank under accession numbers of 5YDF and 5YDE, respectively.

## Electronic supplementary material

Supplementary Information

## References

[1] Carpten JD (2002). HRPT2, encoding parafibromin, is mutated in hyperparathyroidism-jaw tumor syndrome. Nature genetics.

[2] Newey PJ, Bowl MR, Thakker RV (2009). Parafibromin - functional insights. Journal of Internal Medicine.

[3] Newey PJ, Bowl MR, Cranston T, Thakker RV (2010). Cell division cycle protein 73 homolog (CDC73) mutations in the hyperparathyroidism-jaw tumor syndrome (HPT-JT) and parathyroid tumors. Human mutation.

[4] Chen Y (2016). CDC73 gene mutations in sporadic ossifying fibroma of the jaws. Diagnostic pathology.

[5] Gill AJ (2014). Understanding the genetic basis of parathyroid carcinoma. Endocrine pathology.

[6] Zhao J (2007). Sporadic human renal tumors display frequent allelic imbalances and novel mutations of the HRPT2 gene. Oncogene.

[7] Teh BT (1996). Autosomal dominant primary hyperparathyroidism and jaw tumor syndrome associated with renal hamartomas and cystic kidney disease: linkage to 1q21–q32 and loss of the wild type allele in renal hamartomas. The Journal of clinical endocrinology and metabolism.

[8] Szabo J (1995). Hereditary hyperparathyroidism-jaw tumor syndrome: the endocrine tumor gene HRPT2 maps to chromosome 1q21-q31. American journal of human genetics.

[9] Bricaire L (2013). Frequent large germline HRPT2 deletions in a French National cohort of patients with primary hyperparathyroidism. The Journal of clinical endocrinology and metabolism.

[10] Cetani F (2013). CDC73 mutational status and loss of parafibromin in the outcome of parathyroid cancer. Endocrine connections.

[11] Wang PF (2008). Parafibromin, a component of the human PAF complex, regulates growth factors and is required for embryonic development and survival in adult mice. Molecular and Cellular Biology.

[12] Xu Y (2017). Architecture of the RNA polymerase II-Paf1C-TFIIS transcription elongation complex. Nature communications.

[13] Shi X (1996). Paf1p, an RNA polymerase II-associated factor in Saccharomyces cerevisiae, may have both positive and negative roles in transcription. Mol Cell Biol.

[14] Kim J, Guermah M, Roeder RG (2010). The Human PAF1 Complex Acts in Chromatin Transcription Elongation Both Independently and Cooperatively with SII/TFIIS. Cell.

[15] Chu X (2013). Structural insights into Paf1 complex assembly and histone binding. Nucleic Acids Res.

[16] Tomson BN, Arndt KM (2013). The many roles of the conserved eukaryotic Paf1 complex in regulating transcription, histone modifications, and disease states. Biochimica Et Biophysica Acta-Gene Regulatory Mechanisms.

[17] Woodard GE (2005). Parafibromin, product of the hyperparathyroidism-jaw tumor syndrome gene HRPT2, regulates cyclin D1/PRAD1 expression. Oncogene.

[18] Yang Y-J, Han J-W, Youn H-D, Cho E-J (2010). The tumor suppressor, parafibromin, mediates histone H3 K9 methylation for cyclin D1 repression. Nucleic Acids Research.

[19] Lin L, Zhang JH, Panicker LM, Simonds WF (2008). The parafibromin tumor suppressor protein inhibits cell proliferation by repression of the c-myc proto-oncogene. Proceedings of the National Academy of Sciences of the United States of America.

[20] Mosimann C, Hausmann G, Basler K (2006). Parafibromin/Hyrax activates Wnt/Wg target gene transcription by direct association with beta-catenin/Armadillo. Cell.

[21] Takahashi A (2011). SHP2 Tyrosine Phosphatase Converts Parafibromin/Cdc73 from a Tumor Suppressor to an Oncogenic Driver. Molecular Cell.

[22] Kikuchi I (2016). Dephosphorylated parafibromin is a transcriptional coactivator of the Wnt/Hedgehog/Notch pathways. Nature communications.

[23] Mosimann C, Hausmann G, Basler K (2009). The role of Parafibromin/Hyrax as a nuclear Gli/Ci-interacting protein in Hedgehog target gene control. Mechanisms of development.

[24] Amrich CG (2012). Cdc73 subunit of Paf1 complex contains C-terminal Ras-like domain that promotes association of Paf1 complex with chromatin. The Journal of biological chemistry.

[25] Chen H (2012). Crystallographic analysis of the conserved C-terminal domain of transcription factor Cdc73 from Saccharomyces cerevisiae reveals a GTPase-like fold. Acta crystallographica. Section D, Biological crystallography.

[26] Hahn MA, Marsh DJ (2005). Identification of a functional bipartite nuclear localization signal in the tumor suppressor parafibromin. Oncogene.

[27] Hahn MA, Marsh DJ (2007). Nucleolar localization of parafibromin is mediated by three nucleolar localization signals. FEBS letters.

[28] Pazienza, V. *et al*. Identification and Functional Characterization of Three NoLS (Nucleolar Localisation Signals) Mutations of the CDC73 Gene. *PloS one* **8**, doi:10.1371/journal.pone.0082292 (2013). 10.1371/journal.pone.0082292 PMC3855386 24340015

[29] Cetani F (2007). Different somatic alterations of the HRPT2 gene in a patient with recurrent sporadic primary hyperparathyroidism carrying an HRPT2 germline mutation. Endocr Relat Cancer.

[30] Masi G (2014). Characterization of a new CDC73 missense mutation that impairs Parafibromin expression and nucleolar localization. PloS one.

[31] Rozenblatt-Rosen O (2005). The parafibromin tumor suppressor protein is part of a human Paf1 complex. Molecular and Cellular Biology.

[32] Emmott E, Hiscox JA (2009). Nucleolar targeting: the hub of the matter. Embo Reports.

[33] Pederson T, Tsai RYL (2009). In search of nonribosomal nucleolar protein function and regulation. Journal of Cell Biology.

[34] Walls, G. V. *et al*. Mice deleted for cell division cycle 73 gene develop parathyroid and uterine tumours: model for the hyperparathyroidism-jaw tumour syndrome. *Oncogene*, doi:10.1038/onc.2017.43 (2017). 10.1038/onc.2017.43 PMC5472200 28288139

[35] Iwata T, Mizusawa N, Taketani Y, Itakura M, Yoshimoto K (2007). Parafibromin tumor suppressor enhances cell growth in the cells expressing SV40 large T antigen. Oncogene.

[36] Kim J, Roeder RG (2009). Direct Bre1-Paf1 complex interactions and RING finger-independent Bre1-Rad6 interactions mediate histone H2B ubiquitylation in yeast. The Journal of biological chemistry.

[37] Wood A, Schneider J, Dover J, Johnston M, Shilatifard A (2003). The Paf1 complex is essential for histone monoubiquitination by the Rad6-Bre1 complex, which signals for histone methylation by COMPASS and Dot1p. The Journal of biological chemistry.

[38] Bradley KJ (2007). Parafibromin is a nuclear protein with a functional monopartite nuclear localization signal. Oncogene.

[39] Minor W, Cymborowski M, Otwinowski Z, Chruszcz M (2006). HKL-3000: the integration of data reduction and structure solution - from diffraction images to an initial model in minutes. Acta Crystallographica Section D-Biological Crystallography.

[40] Adams PD (2010). PHENIX: a comprehensive Python-based system for macromolecular structure solution. Acta crystallographica. Section D, Biological crystallography.

[41] The CCP4 suite: programs for protein crystallography. *Acta crystallographica. Section D, Biological crystallography* **50**, 760-763, doi:10.1107/s0907444994003112 (1994). 10.1107/S0907444994003112 15299374

[42] Emsley P, Lohkamp B, Scott WG, Cowtan K (2010). Features and development of Coot. Acta crystallographica. Section D, Biological crystallography.

[43] DeLano WL, Brunger AT (1994). Helix packing in proteins: prediction and energetic analysis of dimeric, trimeric, and tetrameric GCN4 coiled coil structures. Proteins.

