# Supplementary material for: Crystal structure of the N-terminal domain of human CDC73 and its implications for the hyperparathyroidism-jaw tumor (HPT-JT) syndrome
Source: Sci Rep. 2017 Nov 15;7:15638. doi: 10.1038/s41598-017-15715-9 (PMC5688130; doi:10.1038/s41598-017-15715-9)
Supplement: Supplementary file 1 — Supplementary Information [file 41598_2017_15715_MOESM1_ESM.pdf]

# Supplementary Information

# Crystal structure of the N-terminal domain of human CDC73 and its implications for the hyperparathyroidism-jaw tumor (HPT-JT) syndrome

Wei Sun[1], Xiao-Lin Kuang[1,2], Yan-Ping Liu[1], Li-Fei Tian[1],
Xiao-Xue Yan[1,4] & Wenqing Xu[1,3,4]

1. National Laboratory of Biomacromolecules, CAS Center for Excellence in Biomacromolecules, Institute of Biophysics, Chinese Academy of Sciences, Beijing 100101, P. R. China.
2. College of Life Sciences, University of Chinese Academy of Sciences, Beijing 100049, China.
3. Department of Biological Structure, University of Washington, Seattle, Washington 98195, USA.
4. Corresponding authors:
   Xiaoxue Yan: snow@ibp.ac.cn
   Wenqing Xu: wxu@u.washington.edu

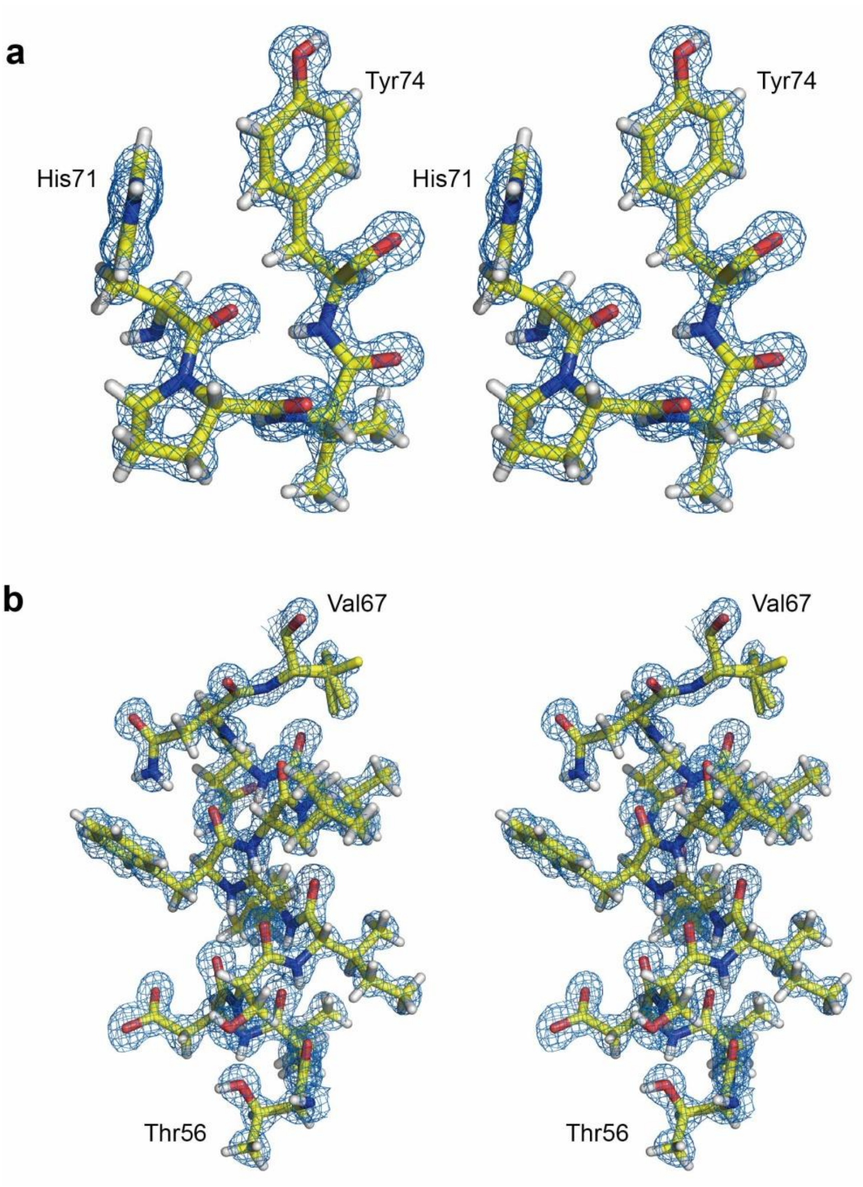


**Supplementary Figure S1.** Electron density map of two typical regions of CDC73(1-111) are shown in panel a and b, respectively. The map was calculated with 2*Fo*-*Fc* coefficients and contoured at 1.5σ as a blue mesh.

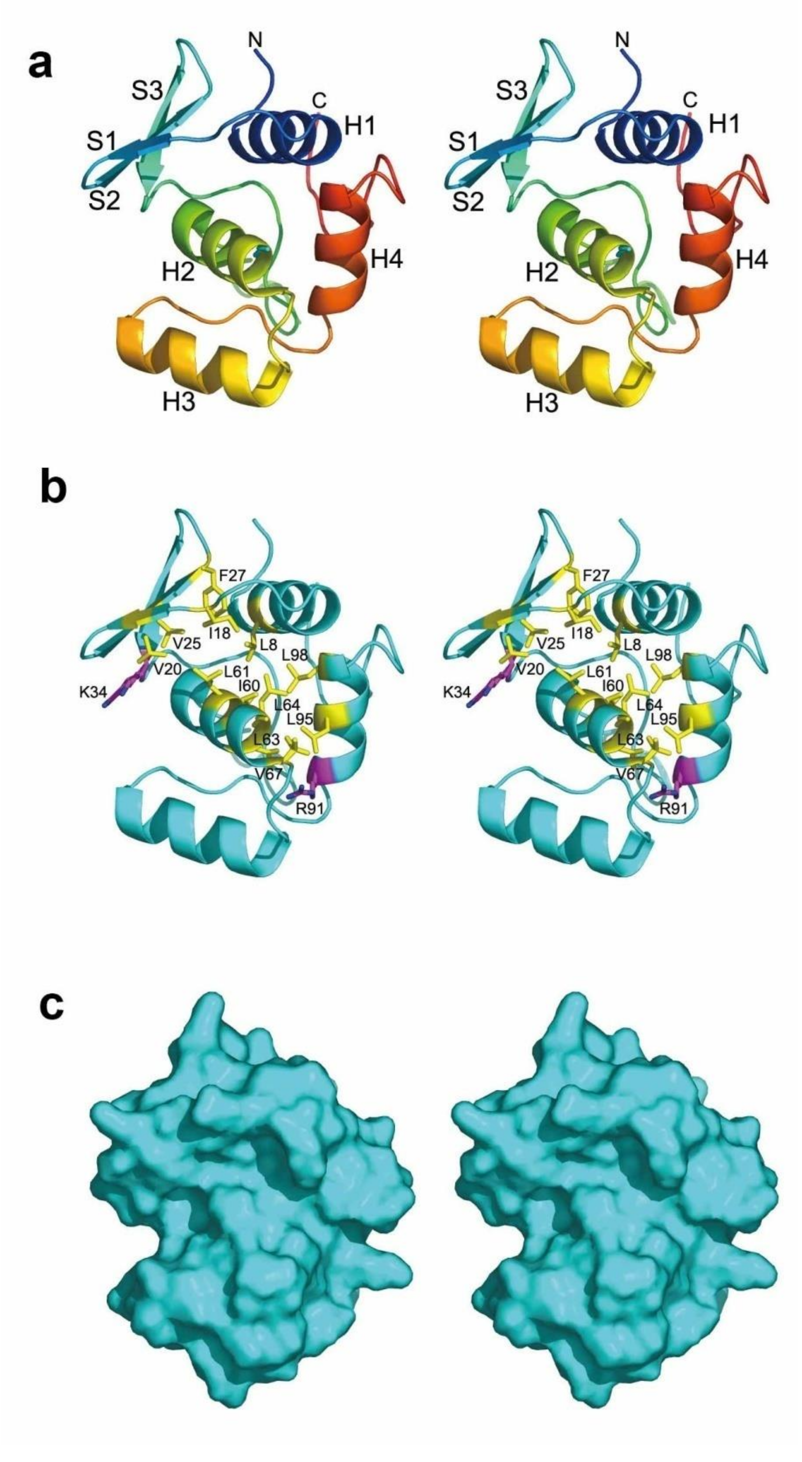


**Supplementary Figure S2.** Stereo views of CDC73-NTD. **(a)** Stereo views of the overall structure. **(b)** Stereo views of the structure with K34, R91 and residues on the hydrophobic groove shown in sticks. **(c)** Stereo views of the surface. All the views are in the same orientation.

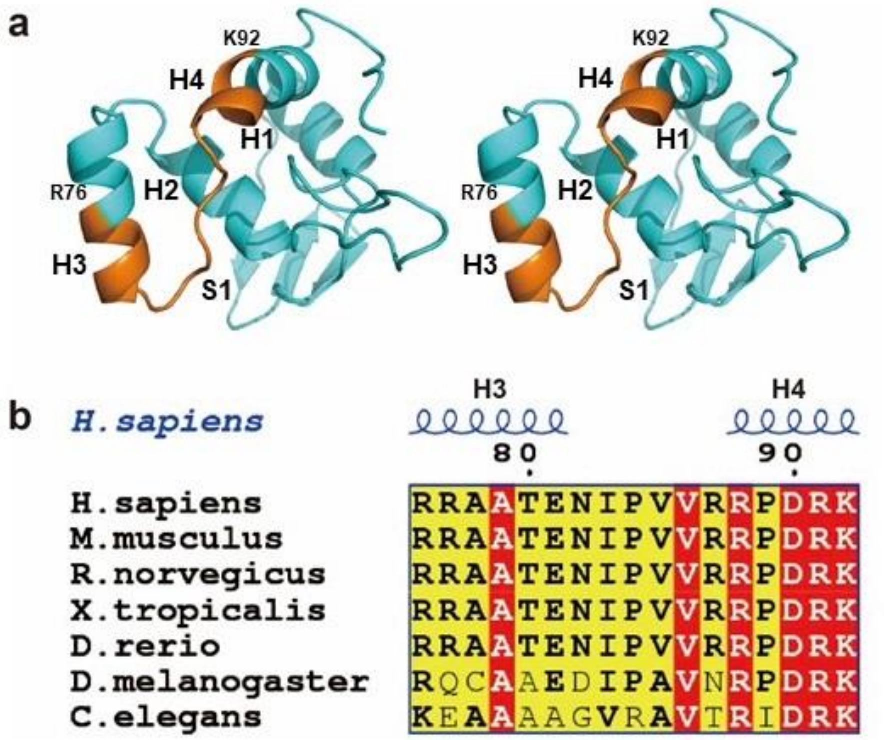


**Supplementary Figure S3.** A proposed nucleolar localization signal (NoLS) in CDC73-NTD. **(a)** Stereo illustration to show that the proposed NoLS forms a surface patch of CDC73-NTD. Residues corresponding to this NoLS, spanning from H3 to H4, are shown in orange. The curved CDC73-NTD hydrophobic groove, formed by the helices H1, H2, H4 and the S1 β-strand, is roughly located in the “back side” of this illustrated structure. **(b)** This proposed NoLS is conserved among species.

**Supplementary Table S1. Human CDC73 variants with explicit clinical significance.**

All these variants, except three missense ones (referenced), are from the ClinVar database. The abbreviations of the clinical manifestation “Condition” are as follows: HPT-JT: hyperparathyroidism-jaw tumor syndrome; FIHP: familial isolated primary hyperparathyroidism; PTC: parathyroid carcinoma; PTA: parathyroid adenoma; CC: clear cell renal carcinoma; OF: ossifying fibroma; N. P.: Not provided.

| Class | Number | Variation | Condition(s) | Clinical significance |
|---|---|---|---|---|
| Missense | 1 | CDC73:c.3G>A (p.Met1Ile) | HPT-JT | Pathogenic |
| | 2[1] | CDC73:c.100A>C (p.Lys34Gln) | CC | Pathogenic |
| | 3[2] | CDC73:c.179T>A (p.Ile60Asn) | OF | Pathogenic |
| | 4 | CDC73:c.191T>C (p.Leu64Pro) | HPT-JT | Pathogenic |
| | 5[3] | CDC73:c.272G>C (p.Arg91Pro) | FIHP, PTA | Pathogenic |
| | | | | |
| Nonsense | 1 | CDC73:c.25C>T (p.Arg9Ter) | HPT-JT | Pathogenic |
| | 2 | CDC73:c.85G>T (p.Glu29Ter) | N.P. | Pathogenic |
| | 3 | CDC73:c.109A>T (p.Lys37Ter) | N.P. | Pathogenic |
| | 4 | CDC73:c.128G>A (p.Trp43Ter) | PTC | Pathogenic |
| | 5 | CDC73:c.162C>G (p.Tyr54Ter) | PTC | Pathogenic |
| | 6 | CDC73:c.226C>T (p.Arg76Ter) | N.P. | Pathogenic |
| | 7 | CDC73:c.355C>T (p.Gln119Ter) | N.P. | Pathogenic |
| | | | | |
| Frameshift | 1 | CDC73:c.4delG (p.Ala2Argfs) | PTC | Pathogenic |
| | 2 | CDC73:c.8_10delACGinsCT (p.Asp3Alafs) | OF | Pathogenic |
| | 3 | CDC73:c.13_16delCTTA (p.Leu5Alafs) | OF | Pathogenic |
| | 4 | CDC73:c.53delT (p.Ile18Metfs) | PTA | Pathogenic |
| | 5 | CDC73:c.85delG (p.Glu29Serfs) | PTC | Pathogenic |
| | 6 | CDC73:c.245delA (p.Asn82Ilefs) | PTC | Pathogenic |
| | 7 | CDC73:c.455_456delGA (p.Arg152Ilefs) | PTC | Pathogenic |
| | 8 | CDC73:c.679_680insAG (p.Arg227Lysfs) | HPT-JT, PTC | Pathogenic |
| | 9 | CDC73:c.687_688dupAG (p.Val230Glufs) | PTC, N.P. | Pathogenic |
| | 10 | CDC73:c.723_725delAGGinsC (p.Gly242Lysfs) | N.P. | Pathogenic |
| | 11 | CDC73:c.766_767delGT (p.Val256Lysfs) | PTC | Pathogenic |
| | | | | |

| | | | | |
|---|---|---|---|---|
| Delins | 1 | CDC73:c.13_30del18 (p.Leu5_Gln10del) | PTC | Pathogenic |
| | 2 | CDC73:c.237+28_237+31delTCTA | PTC | Benign |
| | 3 | CDC73:c.729+50_729+51delAG | PTC | Benign |
| | 4 | CDC73, 4-BP DEL, 685AGAG | PTC, PTA | Pathogenic |
| | 5 | CDC73, 2-BP DEL | HPT-JT | Pathogenic |
| | 6 | CDC73, 1-BP INS, 373A | PTC | Pathogenic |
| | 7 | CDC73, 41-BP DUP/INS | HPT-JT | Pathogenic |
| | 8 | CDC73, 41-BP DUP/INS | HPT-JT | Pathogenic |
| | | | | |
| Splice site | 1 | CDC73:c.131+1G>A | HPT-JT, PTA, N.P. | Pathogenic |
| | 2 | CDC73:c.132-2A>G | HPT-JT | Likely pathogenic |
| | 3 | CDC73:c.237+1G>C | PTC | Pathogenic |
| | 4 | CDC73:c.237+1G>T | HPT-JT, HPT-JT | Pathogenic |
| | 5 | CDC73:c.238-2A>T | N.P. | Pathogenic |
| | 6 | CDC73:c.238-1G>A | HPT-JT | Pathogenic |
| | 7 | CDC73:c.423+1G>A | N.P. | Pathogenic |
| | 8 | CDC73:c.729+1G>T | PTC | Likely pathogenic |
| | | | | |
| UTR | 1 | CDC73:c.-95G>A | PTC, HPT-JT, FIHP | Likely benign |
| | 2 | CDC73:c.-11G>A | PTC | Benign |
| | 3 | CDC73:c.-10G>T | PTC, HPT-JT, FIHP | Benign/Likely benign |
| | 4 | CDC73:c.*12C>A | PTC, HPT-JT,FIHP | Likely benign |
| | 5 | CDC73:c.*94A>G | PTC, HPT-JT, FIHP | Likely benign |
| | 6 | CDC73:c.*518A>G | PTC, HPT-JT, FIHP | Likely benign |
| | 7 | CDC73:c.*580A>T | PTC, HPT-JT, FIHP | Likely benign |
| | 8 | CDC73:c.*1083A>T | PTC, HPT-JT, FIHP | Likely benign |
| | 9 | CDC73:c.*1251A>G | PTC, HPT-JT, FIHP | Likely benign |
| | 10 | CDC73:c.*1267A>G | PTC, HPT-JT, FIHP | Likely benign |
| | 11 | CDC73:c.*1365T>C | PTC, HPT-JT, FIHP | Likely benign |
| | 12 | CDC73:c.*1472T>G | PTC, HPT-JT, FIHP | Likely benign |
| | 13 | CDC73:c.*1499T>A | PTC, HPT-JT, FIHP | Likely benign |
| | 14 | CDC73:c.*1761T>C | PTC, HPT-JT, FIHP | Likely benign |
| | 15 | CDC73:c.*2085G>C | PTC, HPT-JT, FIHP | Likely benign |
| | 16 | CDC73:c.*2304T>G | PTC, HPT-JT, FIHP | Likely benign |
| | 17 | CDC73:c.*2420A>C | PTC, HPT-JT, FIHP | Likely benign |
| | 18 | CDC73:c.*2427T>C | PTC, HPT-JT, FIHP | Likely benign |
| | 19 | CDC73:c.*2677G>A | PTC, HPT-JT, FIHP | Likely benign |
| | 20 | CDC73:c.*2950A>G | PTC, HPT-JT, FIHP | Likely benign |
| | 21 | CDC73:c.*2962T>C | PTC, HPT-JT, FIHP | Likely benign |

| | | | | |
|---|---|---|---|---|
| | 22 | CDC73:c.*3266A>G | PTC, HPT-JT, FIHP | Likely benign |
| | 23 | CDC73:c.*3419T>A | PTC, HPT-JT, FIHP | Likely benign |
| | 24 | CDC73:c.*3507G>T | PTC, HPT-JT, FIHP | Likely benign |
| | 25 | CDC73:c.*3524A>G | PTC, HPT-JT, FIHP | Likely benign |
| | 26 | CDC73:c.*3641G>A | PTC, HPT-JT, FIHP | Likely benign |
| | 27 | CDC73:c.*3786T>C | PTC, HPT-JT, FIHP | Likely benign |
| | 28 | CDC73:c.*3786T>A | PTC, HPT-JT, FIHP | Benign |
| | 29 | CDC73:c.*3811_*3813delTTG | PTC, HPT-JT, FIHP | Likely benign |

**References for Table S1:**


1. Zhao, J. et al. Sporadic human renal tumors display frequent allelic imbalances and novel mutations of the HRPT2 gene. *Oncogene* **26,** 3440-3449 (2007).
2. Masi, G. et al. Characterization of a new CDC73 missense mutation that impairs Parafibromin expression and nucleolar localization. *PLoS One* **9,** e97994 (2014).
3. Cetani, F. et al. Different somatic alterations of the HRPT2 gene in a patient with recurrent sporadic primary hyperparathyroidism carrying an HRPT2 germline mutation. *Endocr Relat Cancer* **14,** 493-499 (2007).

**Supplementary Table S2. Closest structural fold of CDC73-NTD from DALI search.** Only the first 25 hits are shown.

| No. | Chain ID | Z score | r.m.s.d. | Description |
|---|---|---|---|---|
| 1 | 4yrd-A | 3.9 | 3.7 | Capsular polysaccharide synthesis enzyme CAP5F |
| 2 | 3st7-A | 3.6 | 3.6 | Capsular polysaccharide synthesis enzyme CAP5F |
| 3 | 4yrd-B | 3.6 | 3.7 | Capsular polysaccharide synthesis enzyme CAP5F |
| 4 | 3vhr-A | 3.3 | 3.7 | Capsular polysaccharide synthesis enzyme CAP5F |
| 5 | 4xvn-A | 2.8 | 1.9 | Small terminase |
| 6 | 4fcy-B | 2.8 | 2.3 | Transposase |
| 7 | 2fu4-A | 2.5 | 3.7 | Ferric uptake regulation protein |
| 8 | 4xvn-D | 2.5 | 2.1 | Small terminase |
| 9 | 4fcy-A | 2.5 | 2.3 | Transposase |
| 10 | 2m45-A | 2.5 | 3.3 | Minichromosome maintenance protein MCM |
| 11 | 2w57-A | 2.3 | 3.8 | Ferric uptake regulation Protein |
| 12 | 3ehe-A | 2.3 | 3.5 | UDP-glucose 4-epimerase |
| 13 | 3c46-A | 2.3 | 4.3 | Virion RNA polymerase |
| 14 | 2y9z-A | 2.2 | 4.1 | Imitation switch protein 1 |
| 15 | 1pdn-C | 2.2 | 1.9 | DNA-paired domain complex |
| 16 | 4iof-A | 2.2 | 2.9 | Insulin-degrading enzyme |
| 17 | 3dpl-C | 2.2 | 4.9 | Cullin-5 |
| 18 | 4bkx-A | 2.2 | 4.2 | Metastasis-associated protein MTA1 |
| 19 | 3elk-A | 2.2 | 2.9 | Putative transcriptional regulator TA0346 |
| 20 | 4j2o-C | 2.1 | 3.6 | UDP-N-acetylglucosamine 4,6- dehydratase/5-epimerase |
| 21 | 6pax-A | 2.1 | 2.6 | Homeobox protein PAX-6 |
| 22 | 3hm5-A | 2.1 | 3.0 | DNA methyltransferase 1-associated protein 1 |
| 23 | 1mwk-A | 2.1 | 3.2 | PARM |
| 24 | 3c3w-A | 2.1 | 3.5 | Two component transcriptional regulatory protein |
| 25 | 2hyt-A | 2.1 | 3.0 | TETR-family transcriptional regulator |
